# Carnivore and Animal-Based Diets in Sport: A Critical Evaluation of Current Evidence and Future Perspectives for Precision Nutrition

**DOI:** 10.3390/nu18060998

**Published:** 2026-03-21

**Authors:** Zbigniew Waśkiewicz

**Affiliations:** Institute of Sport Sciences, Academy of Physical Education in Katowice, 40-065 Katowice, Poland; z.waskiewicz@awf.katowice.pl

**Keywords:** carnivore diet, animal-based diet, sports nutrition, low-carbohydrate diets, ketogenic diet, athletic performance, metabolic adaptation, gut microbiota, dietary restriction, precision nutrition

## Abstract

The increasing popularity of carnivore and animal-based diets among athletes has generated substantial interest, despite limited direct scientific evidence supporting their efficacy and safety in sport-specific contexts. This narrative review critically evaluates the current evidence and examines the physiological, performance, and health-related implications of these dietary models in athletic populations. These dietary models, characterized by the partial or complete exclusion of plant-derived foods, are often promoted on the basis of mechanistic arguments, anecdotal reports, and extrapolations from research on ketogenic and very low-carbohydrate diets. However, their physiological relevance, long-term health implications, and compatibility with the demands of athletic training remain poorly defined. This narrative review provides a critical perspective on the current evidence related to carnivore and animal-based diets in sport, integrating findings from studies on low-carbohydrate, ketogenic, high-protein, and elimination-based dietary patterns. The analysis focuses on metabolic adaptations, body composition, exercise performance, gastrointestinal function, micronutrient adequacy, hormonal responses, and potential long-term health risks. Particular attention is given to the distinction between metabolic adaptations and functional performance outcomes, as well as to the high interindividual variability in dietary responses. The available evidence suggests that while carbohydrate restriction may induce specific metabolic adaptations, such as increased fat oxidation, these changes do not consistently translate into improved performance, particularly in high-intensity or high-volume training contexts. Moreover, the highly restrictive nature of carnivore and animal-based diets raises concerns about micronutrient deficiencies, alterations in the gut microbiota, changes in the lipid profile, and potential effects on eating behaviours, particularly in competitive athletic populations. Given the absence of well-controlled, long-term intervention studies in athletes, carnivore and animal-based diets cannot currently be recommended as safe or optimal nutritional strategies for sports performance. Rather than representing viable alternatives to established sports nutrition guidelines, these dietary models may be better understood as experimental or short-term tools within highly controlled research or diagnostic frameworks. Future research should prioritize rigorous, sport-specific study designs, long-term safety outcomes, and personalized approaches that account for individual metabolic and physiological variability.

## 1. Introduction

Sports nutrition has been a central research domain within sports science for decades, and its importance for exercise performance, training adaptations, and long-term health has been unequivocally confirmed by numerous expert reports and practical guidelines [1]. For most of the twentieth and early twenty-first centuries, the dominant paradigm in sports nutrition was based on high-carbohydrate models, grounded in the pivotal role of muscle glycogen in sustaining exercise at moderate to high intensities [2]. These recommendations were formally reflected in position stands issued by international nutrition and sports organizations [3].

With the development of nutritional periodization concepts, increasing attention has been directed toward the need for more flexible adjustment of macronutrient intake to training demands and seasonal phases [4]. Within this framework, interest emerged in strategies involving periodic carbohydrate restriction, with the primary aim of modulating metabolic adaptations rather than inducing a permanent shift in dietary patterns [5].

Early investigations into physiological adaptation to very low-carbohydrate diets demonstrated that prolonged restriction of glucose availability leads to substantial alterations in energy metabolism, including a marked increase in fatty acid oxidation capacity [6]. Cross-sectional analyses of keto-adapted ultra-endurance athletes have demonstrated markedly elevated rates of fat oxidation compared with high-carbohydrate athletes, without clear evidence of superior performance outcomes [7]. Subsequent studies confirmed that athletes adapted to ketogenic diets can achieve exceptionally high rates of fat oxidation during exercise [8].

At the same time, a growing body of evidence highlighted important functional limitations of such adaptations during high-intensity exercise. In elite endurance athletes, short-term adherence to a low-carbohydrate, high-fat (LCHF) diet impaired exercise economy and failed to improve performance [9]. These findings were corroborated by studies of elite endurance athletes, which showed that restricted carbohydrate availability negatively affected competitive performance [10]. Systematic reviews further indicate that performance responses to ketogenic diets are highly variable and strongly dependent on the specific sporting context [11].

Importantly, the performance implications of carbohydrate-restricted diets appear to depend strongly on the physiological demands of the specific sporting discipline. In sports characterized by prolonged submaximal exercise, such as ultra-endurance running or long-distance cycling, increased reliance on lipid oxidation may theoretically confer metabolic advantages. In contrast, disciplines requiring repeated high-intensity efforts, rapid accelerations, or high glycolytic flux—such as team sports, middle-distance events, or combat sports—are likely to be negatively affected by chronically reduced carbohydrate availability. In some applied contexts, particularly in weight-category sports such as wrestling, combat sports, or powerlifting, short-term carbohydrate restriction may also be used as a practical strategy to facilitate rapid reductions in body mass prior to competition. However, such applications are typically transient and should not be interpreted as evidence supporting the long-term use of ketogenic or carnivore-type dietary models for performance enhancement.

Against this background, recent years have seen growing interest in extremely restrictive dietary models, such as the carnivore diet and the broader animal-based approach. These models are characterized by a dominant reliance on animal-derived foods and a substantial reduction or complete elimination of plant-based foods [12]. Despite their increasing popularity within athletic communities, empirical data on their effects on health and performance remain very limited and largely observational [13].

In scientific practice, the evaluation of carnivore and animal-based diets relies largely on extrapolation from studies on ketogenic and very low-carbohydrate diets, which introduces significant interpretative limitations [14]. To date, no randomized controlled trials have directly examined carnivore diets in athletic populations. Existing scientific discussions have largely extrapolated from research on ketogenic and very low-carbohydrate dietary models [15], which introduces important interpretative limitations. Concurrently, the development of precision nutrition concepts and individualized dietary interventions suggests that responses to restrictive dietary patterns may be strongly influenced by individual characteristics [16].

Several biological and behavioral factors may contribute to this interindividual variability. These include genetic polymorphisms affecting lipid and carbohydrate metabolism, baseline insulin sensitivity, habitual dietary intake, gut microbiome composition, and the athlete’s training status and metabolic flexibility. Emerging research in nutrigenomics and metabolomics suggests that these factors may substantially influence both tolerance to carbohydrate restriction and the magnitude of resulting metabolic adaptations. Consequently, identical dietary interventions may elicit markedly different physiological responses across individuals, further complicating the formulation of universal dietary recommendations.

Research in nutrigenomics and metabolomics indicates that interindividual differences in metabolic regulation may determine both tolerance to specific dietary strategies and their potential functional outcomes [17]. Additionally, it has been shown that glycemic responses to identical dietary stimuli can vary substantially among individuals, undermining the validity of universal dietary recommendations [18]. In this context, carnivore and animal-based diets represent not only a source of controversy but also a potential framework for exploring the limits of human metabolic adaptation [19].

The present review aims to critically synthesize the available literature on carnivore- and animal-based diets in sport, considering physiological mechanisms, potential benefits, and health-related limitations. This work is conceived as a narrative review, intended to identify research gaps and establish interpretative frameworks for future empirical studies rather than to formulate normative dietary recommendations. To facilitate interpretation of this complex topic, the review is organized into several sections addressing (1) conceptual definitions of carnivore and animal-based diets, (2) physiological mechanisms and exercise-related adaptations, (3) potential effects on body composition and metabolic health, and (4) future research perspectives within the framework of precision nutrition.

## 2. Methods

### 2.1. Study Design and Methodological Rationale

This review was designed as a narrative review to critically synthesize the available evidence on carnivore- and animal-based diets in the context of sport and physical activity. The choice of this methodological approach stems from the limited number of empirical studies examining these dietary models in athletic populations, as well as the substantial heterogeneity of the available indirect evidence [20]. Under such conditions, guidelines that apply formal systematic review procedures, such as PRISMA, could yield conclusions that appear methodologically precise but are conceptually problematic [21].

The narrative nature of the review enables integration of findings from diverse research domains, including exercise physiology, energy metabolism, sports nutrition, and metabolic health. This approach is particularly justified for carnivore- and animal-based diets, for which direct evidence is sparse and much of the relevant knowledge derives from studies of very low-carbohydrate, ketogenic, and high-protein diets [15]. Conscious extrapolation of data was therefore employed as an interpretative tool rather than as evidential support, allowing transparency regarding the boundaries of inference [22].

Literature selection was based on peer-reviewed publications from established scientific databases and encompassed both interventional studies and narrative and systematic reviews. Particular emphasis was placed on studies examining metabolic adaptations to carbohydrate restriction, the effects of high-protein intake on body composition and health, and the consequences of long-term elimination of plant foods on gastrointestinal function [23]. This strategy enabled broad mapping of the research landscape without artificially constraining the analysis to a single interpretative framework [24]. Animal-dominant elimination models raise distinct nutritional and metabolic considerations that remain insufficiently studied in athletes.

A key methodological principle of the present review was the consistent distinction between direct and indirect evidence. Findings derived from studies on ketogenic, low-carbohydrate, and high-protein diets were treated as comparative material rather than as direct evidence of the efficacy of carnivore and animal-based diets. This distinction is essential to avoid overinterpretation, a recurrent issue in nutrition research [25]. The analysis also accounted for well-documented limitations of self-reported dietary data and pattern classification, including systematic misreporting and definitional heterogeneity [26]. These issues are particularly relevant in the context of elimination diets, which lack standardization and are defined heterogeneously across studies [27]. Consequently, interpretation of findings was guided by a principle of caution, with an emphasis on identifying evidentiary limitations. Finally, the literature analysis focused on identifying research gaps and areas requiring further empirical investigation rather than on generating prescriptive dietary recommendations. This approach aligns with contemporary perspectives on interpreting nutritional data in the context of complex dietary interventions [28]. The analytical framework of the present review was explicitly structured around the distinction between metabolic adaptations and functional performance outcomes. Metabolic changes induced by dietary interventions, such as alterations in substrate utilization, insulin response, or fat oxidation capacity, were consistently interpreted separately from their practical relevance for exercise performance, training quality, and competitive demands. This distinction was adopted to avoid conflating physiological mechanisms with functional efficacy, a common source of overinterpretation in sports nutrition research, particularly in the context of carbohydrate-restricted dietary models.

### 2.2. Literature Identification and Selection Strategy

To enhance transparency while preserving the narrative character of the review, a structured but non-systematic literature identification strategy was applied. The literature search was conducted across major scientific databases commonly used in sports and nutrition research, including PubMed, Scopus, and Web of Science, without formal protocol registration or PRISMA-based screening procedures. Searches focused on peer-reviewed publications addressing low-carbohydrate, ketogenic, high-protein, elimination-based, carnivore, and animal-based dietary patterns in the context of exercise, sport, metabolic adaptation, and health outcomes. Priority was given to experimental studies, systematic and narrative reviews, and position or consensus papers published in established journals in sports nutrition, exercise physiology, and metabolic research. Given the absence of randomized controlled trials directly examining carnivore or animal-based diets in athletic populations, studies investigating closely related dietary models, such as very-low-carbohydrate or ketogenic diets, were deliberately included as indirect comparative evidence. Such studies were not treated as direct support for carnivore or animal-based diets, but rather as contextual material informing potential physiological mechanisms and functional limitations. No rigid temporal cut-off was imposed; however, greater emphasis was placed on literature published within the last 15 years, alongside seminal earlier studies that established foundational concepts in substrate metabolism, muscle glycogen regulation, and exercise performance. The selection process was guided by relevance, methodological quality, and conceptual contribution rather than by exhaustive coverage, in line with the interpretative objectives of a critical narrative review.

To improve methodological transparency, the literature search combined terms related to dietary models, including “carnivore diet,” “animal-based diet,” “ketogenic diet,” and “low-carbohydrate diet,” with terms related to exercise and sport, such as “athlete,” “exercise performance,” “endurance,” “strength,” “metabolism,” and “sports nutrition.” The search was limited to peer-reviewed literature published in English. Because of the narrative nature of the review and the scarcity of direct studies on carnivore diets in athletes, no formal inclusion and exclusion framework was applied beyond relevance to sport, metabolic adaptation, performance, and health outcomes. A summary of the general search structure, including core keywords and database coverage, is provided in Supplementary Table S1.

## 3. Definitions and Characteristics of Carnivore and Animal-Based Diets

Because these dietary models are frequently conflated in both scientific and popular discourse, it is essential to clearly distinguish between carnivore diets, broader animal-based dietary patterns, and ketogenic or very-low-carbohydrate diets. Although these approaches may share certain metabolic features, they differ substantially in dietary composition, degree of plant food exclusion, and potential physiological consequences. The terms “carnivore diet” and “animal-based diet” are used ambiguously in both the scientific literature and nutritional practice, which constitutes a major source of interpretive difficulty when assessing their potential relevance in sport [29]. The absence of standardized definitions leads to these terms being used interchangeably or overly simplistically, even though, in practice, they refer to different degrees of dietary restriction and distinct nutritional assumptions [30].

The carnivore diet is generally characterized as a dietary model composed almost exclusively of animal-derived foods, including meat, fish, eggs, and, in some variants, selected dairy products. Although no standardized scientific definition currently exists, available survey-based evidence indicates that individuals who identify with a carnivore diet typically report a complete or near-complete elimination of plant-based foods [13]. In contrast to conventional low-carbohydrate diets, in which carbohydrate restriction is intentionally implemented to induce specific metabolic adaptations, the carnivore diet functions primarily as an elimination-based model. Within this structure, carbohydrate restriction arises from excluding plant-derived foods rather than from a targeted metabolic strategy.

A major methodological challenge is the lack of standardization of the carnivore diet as a research intervention. In practice, substantial heterogeneity is observed among variants based exclusively on ruminant meat, versions that permit eggs and dairy, and approaches that incorporate periodic modifications [13]. This variability complicates comparison across observational findings and precludes treating the carnivore diet as a homogeneous category in scientific analyses.

The animal-based diet lies between the carnivore diet and other low-carbohydrate dietary models. Carbohydrate restriction has been widely proposed as a primary dietary strategy for improving glycemic control and stabilizing insulin dynamics, particularly in metabolically demanding contexts [31]. In this approach, animal-derived foods play a dominant role, while selected plant-based foods are permitted, most commonly fruits or other carbohydrate sources with low levels of antinutritional compounds [32,33]. In contrast to the carnivore diet, the animal-based model does not require complete elimination of plant foods, which may mitigate some of the potential consequences of a long-term absence of dietary fibre [34].

From the perspective of nutritional physiology, carnivore- and animal-based diets should be considered part of a continuum of carbohydrate-restricted strategies rather than distinct metabolic entities [35]. Within this framework, the carnivore diet is the most restrictive elimination-based variant, whereas the animal-based diet falls between ketogenic diets and less restrictive low-carbohydrate models [36].

This positioning has important interpretative implications, as it suggests that the potential physiological effects of these diets should be examined in the context of adaptations documented in research on ketogenic and very low-carbohydrate diets [37]. At the same time, the extreme elimination of plant-based foods characteristic of the carnivore diet introduces additional variables do not present in classical ketogenic diets, including the absence of dietary fibre and phytochemicals [34]. Some versions of animal-based dietary models also recommend restricting seed oils, which are often portrayed within popular dietary narratives as metabolically harmful or pro-inflammatory. However, current scientific evidence does not consistently support these claims. Many plant-derived oils are rich sources of polyunsaturated fatty acids that have been associated with favorable cardiovascular outcomes in epidemiological and clinical research. As a result, the exclusion of seed oils in certain animal-based dietary frameworks appears to reflect theoretical or ideological considerations rather than conclusions derived from a consistent body of experimental evidence.

The lack of unambiguous definitions of carnivore and animal-based diets consequently imposes significant limitations on the comparability of studies and on the formulation of generalized conclusions about their effects on health and athletic performance [15]. For this reason, further analyses of these dietary models require precise specification of the degree of dietary restriction and a conscious consideration of their position within the broader context of low-carbohydrate dietary strategies [38].

Given the conceptual overlap and frequent conflation of carnivore, animal-based, and very low-carbohydrate dietary models in both scientific and popular discourse, a structured comparison is warranted. Table 1 summarizes the defining characteristics of these dietary approaches with respect to food inclusion criteria, macronutrient distribution, metabolic state, and degree of dietary restriction. This overview provides a clear conceptual framework for the mechanistic and performance-related considerations discussed in subsequent sections.

## 4. Physiological Mechanisms and Exercise-Related Adaptations

Considering the physiological mechanisms associated with carnivore- and animal-based diets in sport first requires rejecting narratives that suggest these approaches constitute a qualitatively new category of nutritional intervention. From the perspective of exercise physiology, these strategies intensify and radicalize adaptations already well documented in research on very low-carbohydrate diets rather than introducing fundamentally distinct metabolic pathways [15]. Framing the issue in this way avoids the illusion of novelty and provides a more realistic foundation for interpreting potential effects.

One of the most consistently documented adaptations to carbohydrate restriction is an increased capacity for fatty acid oxidation. Classical studies demonstrated that prolonged carbohydrate restriction leads to profound reorganization of energy metabolism, encompassing changes in both enzymatic activity and hormonal regulation [6]. These adaptations were later confirmed in well-trained endurance athletes, in whom exceptionally high rates of fat oxidation during exercise were observed [8]. In this sense, carnivore and animal-based diets may be viewed as interventions that maximize one extreme of the substrate utilization spectrum.

However, enhanced fat oxidation should not be conflated with improved exercise performance. The capacity to rely more heavily on lipid substrates represents a metabolic shift rather than a functional outcome. In competitive sport, performance is determined by the ability to sustain high rates of ATP turnover under variable and often high-intensity conditions. Fat oxidation, although energetically efficient at lower intensities, is oxygen-costly and slower in ATP resynthesis compared with carbohydrate oxidation [42]. Consequently, the metabolic adaptation frequently cited as a benefit of carbohydrate restriction may simultaneously constrain exercise economy and high-intensity work capacity [9,43]. Framing increased fat oxidation as inherently advantageous therefore represents a category error in the interpretation of exercise physiology.

The relevance of these costs is especially apparent in disciplines that require high relative intensity or the ability to perform repeated accelerations. Reviews of the literature indicate that under such conditions, carbohydrate restriction is associated with impaired performance, even in athletes exhibiting advanced metabolic adaptation [44]. Findings from studies of elite race walkers further confirm that limited carbohydrate availability can adversely affect competitive performance despite increased fat-oxidation capacity [45].

In this context, the concept of metabolic flexibility becomes particularly salient. Metabolic flexibility is the capacity to dynamically switch between energy substrates in response to changes in exercise intensity [46]. Carnivore and animal-based diets, through their extreme reduction of carbohydrate intake, may constrain this flexibility, with direct consequences for the ability to perform complex training loads [47]. From a practical standpoint, this may limit adaptive potential in sports characterized by variable exercise demands.

A central factor underlying these limitations is the availability of muscle glycogen. Classical investigations demonstrated a direct relationship between muscle glycogen availability and the capacity to sustain high-intensity exercise [48]. Contemporary analyses of skeletal muscle energetics confirm that carbohydrate-derived substrates remain essential for maintaining exercise intensity when rapid ATP turnover is required [42]. Contemporary analyses confirm that low muscle glycogen remains a primary limiting factor for performance in high-intensity sports [22]. In this respect, adaptations to carnivore and animal-based diets may enhance certain aspects of metabolism while simultaneously constraining other functionally critical capacities. An additional adaptive dimension associated with these diets arises from their very high protein content. Meta-analyses clearly demonstrate that adequate amino acid availability is essential for stimulating muscle protein synthesis and supporting hypertrophic adaptations [49]. High-protein dietary models may therefore help preserve fat-free mass, even under energy deficit [50]. At the same time, combining high protein intake with carbohydrate restriction increases reliance on gluconeogenesis, which may affect hormonal regulation and overall metabolic load [51].

From a metabolic health perspective, carbohydrate restriction is associated with reduced insulin responses and altered insulin sensitivity [52]. Although such adaptations are often interpreted as beneficial, their functional relevance in athletic populations remains ambiguous, given that high insulin sensitivity is frequently a baseline characteristic of regularly training individuals [53]. As a result, potential metabolic benefits may not translate directly into improvements in athletic performance.

Finally, analysis of exercise-related adaptations cannot overlook the impact of restrictive diets on recovery processes. While some studies suggest alterations in metabolic and oxidative stress markers [41], there is no consistent evidence demonstrating the superiority of carnivore- or animal-based diets for post-exercise recovery [54]. Moreover, evidence from elite endurance athletes indicates that chronic adherence to ketogenic low-carbohydrate models may impair training quality and reduce the capacity to sustain high-intensity workloads [9,40].

Taken together, these observations should be interpreted within the framework of contemporary models of exercise regulation, in which glucose, lipid, and lactate metabolism form an integrated energetic system [55]. From this perspective, carnivore and animal-based diets are neither unequivocally “beneficial” nor “detrimental” but rather shift the balance among different components of the energy system. Their potential relevance in sport therefore depends on the specific sporting context, the structure of training loads, and individual athlete characteristics.

Importantly, enhanced fat oxidation capacity does not necessarily translate into improved exercise performance across all exercise intensities. Evidence from studies examining carbohydrate-restricted dietary strategies suggests that reduced carbohydrate availability may impair performance during high-intensity exercise, where rapid ATP production from glycolytic pathways is essential. These findings indicate that although metabolic adaptations toward increased fat utilization may support prolonged submaximal exercise, they may simultaneously limit the capacity to sustain repeated high-intensity efforts commonly observed in many competitive sports.

## 5. Body Composition, Metabolic Health, and Potential Risks

Evaluation of carnivore- and animal-based diets in sport inevitably shifts the analytical focus from short-term exercise adaptations to body composition and metabolic health. Within this domain, restrictive dietary models are most frequently promoted as potentially “optimal,” particularly in disciplines where reductions in body mass or fat mass are perceived as performance-enhancing [56].

There is little doubt that very low-carbohydrate, high-protein diets facilitate body mass reduction in some individuals, primarily by reducing spontaneous energy intake and by the strong satiety-inducing effects of protein [50]. However, from a sporting perspective, the critical issue is not body mass reduction per se, but rather the preservation or improvement of the lean-to-fat mass ratio. Meta-analyses indicate that high protein intake can support the maintenance of fat-free mass during periods of energy deficit [49], making this aspect of carnivore- and animal-based diets potentially attractive in applied sport settings.

At the same time, it must be clearly emphasized that observed changes in body composition are not unique to elimination-based diets centered on animal-derived foods. Controlled studies demonstrate that fat mass reduction can be achieved with a variety of dietary models, provided an energy deficit is maintained [57]. In this context, the postulated “metabolic advantage” of carnivore and animal-based diets lacks consistent empirical support [31].

A considerably more problematic area concerns the impact of these diets on gastrointestinal function and the gut microbiome. Experimental data unequivocally demonstrate that dietary composition can induce substantial alterations in microbiota structure within a relatively short period [58]. Long-term elimination of plant-based foods is associated with near-complete exclusion of dietary fibre, which plays a central role in maintaining gut microbial diversity [59].

Claims regarding the benefits of carnivore and animal-based diets in sport often extend beyond the currently available empirical evidence. To clearly distinguish among theoretical rationale, indirect findings from related dietary models, and direct experimental support, Table 2 contrasts commonly cited health- and performance-related claims with the actual strength and scope of the supporting literature. In Table 2, the classification of “evidence strength” represents a qualitative interpretative synthesis rather than a formal evidence-grading framework. The categorization was based on the presence or absence of direct empirical studies examining carnivore or animal-based diets, the consistency of findings derived from related dietary models such as ketogenic or very-low-carbohydrate diets, and the methodological robustness of the available literature. Because randomized controlled trials specifically examining carnivore diets in athletic populations are currently lacking, most classifications necessarily rely on indirect evidence and should therefore be interpreted with caution.

Studies suggest that low-fibre diets may reduce populations of bacteria that produce short-chain fatty acids, potentially influencing inflammatory and metabolic regulation [67]. Although some of these changes may be reversible after reintroducing plant foods [68], there is insufficient data to evaluate the long-term consequences of such adaptations in athletes, whose gastrointestinal systems are further challenged by intensive training loads [69]. From a metabolic health perspective, low-carbohydrate diets are sometimes associated with improvements in selected markers, such as glycemic control and reductions in triglyceride concentrations [56]. A central interpretative limitation of the current evidence base is the heavy reliance on data derived from clinical or metabolically compromised populations. Improvements observed in individuals with obesity, insulin resistance, or type 2 diabetes cannot be automatically translated to healthy, well-trained athletes whose baseline metabolic function is already optimized. In such contexts, apparent “benefits” may simply reflect normalization of dysregulated physiology rather than performance-enhancing adaptations. Extrapolating therapeutic effects observed in disease states to high-performance sport risks overstating both the magnitude and relevance of dietary interventions. A key interpretative limitation of the available evidence is that a substantial proportion of studies examining metabolic and health-related effects of carbohydrate-restricted diets have been conducted in clinical or metabolically compromised populations. While these findings provide valuable insights into potential mechanisms, their direct applicability to healthy, well-trained athletes is inherently limited. Differences in baseline insulin sensitivity, training-induced metabolic adaptations, and physiological resilience may substantially modify both the magnitude and direction of dietary effects, underscoring the need for caution when translating clinical nutrition data into sport-specific contexts.

Particular attention should be given to the effects of carnivore and animal-based diets on lipid profiles. While some individuals exhibit neutral changes, others experience substantial increases in LDL cholesterol concentrations [70]. The pronounced interindividual variability observed in lipid responses to carbohydrate-restricted and animal-derived dietary patterns represents an additional interpretative limitation of the current evidence. Group-level analyses may obscure clinically meaningful responses in specific subgroups, including so-called hyper-responders, who exhibit disproportionate elevations in LDL cholesterol despite otherwise favorable metabolic markers. In the absence of stratified analyses or long-term cardiovascular outcome data in athletic populations, population-averaged findings should not be assumed to reflect individual risk profiles. This variability underscores the importance of individual metabolic responsiveness and the need to monitor health parameters when highly restrictive elimination-based dietary models are implemented.

A further concern is the risk of micronutrient inadequacy. Excluding plant foods may lead to low intakes of vitamin C, folate, magnesium, and certain bioactive compounds [71,72]. Although animal-derived foods provide many essential minerals [73], limited dietary diversity increases the risk of subclinical deficiencies, which may be relevant for athlete health and recovery.

Regarding long-term safety, a frequently cited concern is the potential impact of high protein intake on renal function. Data from healthy athletes do not provide clear evidence of adverse effects of a high-protein diet [74]. Nevertheless, studies assessing the long-term effects of carnivore- and animal-based diets under conditions of intensive training are lacking [75].

Finally, the psychological dimension should not be overlooked. Extremely restrictive elimination-based dietary models may affect the relationship with food and promote restrictive eating behaviors, which represent a meaningful risk in elite athletic populations [76]. For this reason, the evaluation of carnivore- and animal-based diets in sport cannot be limited to physiological indices alone; it must incorporate a broader perspective that encompasses mental health and behavioral context.

In addition to potential metabolic adaptations, highly restrictive dietary models such as strict carnivore diets may also raise important health considerations. The near-complete exclusion of plant foods eliminates primary dietary sources of fiber and several micronutrients, which may increase the risk of nutritional imbalances over prolonged periods. In particular, low fiber intake may influence gut microbiota composition and reduce the production of short-chain fatty acids that play an important role in intestinal and metabolic health. Furthermore, the long-term cardiovascular implications of diets characterized by high intake of animal-derived fats and proteins remain insufficiently investigated in athletic populations. These considerations highlight the importance of cautious interpretation and reinforce the need for well-controlled long-term studies evaluating both performance outcomes and health markers.

## 6. Discussion

Discussion of carnivore and animal-based diets in sport reveals a fundamental tension between the appeal of simplified metabolic narratives and the complexity of the physiological demands imposed by modern athletic training. In popular discourse and parts of the scientific literature, there is a tendency to equate isolated metabolic adaptations with overall improvements in performance or health, leading to overgeneralization [15]. Available evidence instead suggests that adaptations induced by carbohydrate restriction are highly context-dependent and cannot be interpreted independently of exercise structure, training intensity, and sport-specific goals [22].

One key source of misunderstanding in this debate is the transfer of conclusions about fat oxidation capacity to assessments of real-world sporting utility. Although increased lipid oxidation is an unequivocal physiological adaptation, its functional relevance is limited in disciplines requiring high relative intensity or frequent pace changes [77]. In this sense, improvements in one metabolic parameter may co-occur with deterioration in other capacities critical for sporting success, which calls into question the validity of unqualified recommendations [41].

A further issue requiring critical reflection is the divergence between markers of metabolic health and indices of athletic performance. In the low-carbohydrate literature, improved glycemic control or enhanced insulin sensitivity is often cited as evidence supporting the use of low-carbohydrate diets [78]. However, in healthy, well-trained athletes, these parameters are often already optimal at baseline, thereby limiting potential functional gains [79]. Consequently, adaptations that benefit metabolic health do not necessarily translate into improved exercise capacity. Another layer of complexity is the substantial interindividual variability in responses to restrictive dietary models. Evidence indicates that both metabolic adaptations and health consequences of low-carbohydrate diets may differ markedly between individuals, even at comparable levels of training status [18]. This variability challenges the rationale for population-level recommendations and suggests that carnivore and animal-based diets may be tolerated by narrow subgroups, but do not constitute a universal nutritional strategy [19]. It should be acknowledged that, as with all narrative reviews, the present synthesis is inherently shaped by interpretative judgment. It does not aim to provide an exhaustive or quantitatively weighted summary of the literature.

Important methodological limitations further constrain the interpretation of the available evidence. Research in sports nutrition relies heavily on self-reported dietary data, which are subject to reporting error and definitional ambiguity in dietary pattern definitions [26]. In the case of carnivore- and animal-based diets, this problem is amplified, as the lack of standardized interventions complicates comparisons across studies and facilitates overinterpretation of isolated observations [31]. Within this context, it is essential to distinguish between short-term adaptations and long-term health consequences. Some putative benefits observed during early phases of carbohydrate restriction may reflect transient metabolic shifts that do not necessarily persist over longer time horizons [80]. The lack of long-term data on athletes following carnivore and animal-based diets currently precludes a robust assessment of safety over a multi-year athletic career [81]. A further critical limitation of the existing evidence base is the short duration of most dietary intervention studies informing the present synthesis. The majority of available trials span weeks rather than months, which restricts interpretation to early or transient metabolic responses and precludes reliable inference regarding long-term health, recovery capacity, or cumulative training adaptations. In athletic populations, where dietary strategies are often implemented chronically across training cycles and competitive seasons, this temporal mismatch represents a substantial constraint on the external validity of current findings.

An important, though often marginalized, issue concerns the influence of restrictive diets on eating behaviors and mental health in athletes. Extremely elimination-based dietary models may foster restrictive behavioral patterns and disrupt the relationship with food, which represents a significant risk in elite sport [82]. From a long-term athlete development perspective, this dimension should be treated as equally important as physiological parameters.

When synthesizing the broader evidence base, it is also necessary to consider the role of media and community narratives in shaping perceptions of carnivore and animal-based diets. The popularity of these dietary models frequently outpaces the scientific evidence, and isolated metabolic mechanisms are sometimes used to justify far-reaching claims [83]. This phenomenon aligns with a wider problem of overinterpretation in nutrition research, in which complex biological interactions are reduced to simplistic messages [84]. The rapid dissemination of simplified dietary narratives through social media, podcasts, and non-academic platforms introduces an additional layer of interpretative bias that is difficult to quantify but highly relevant to applied sport contexts. Claims surrounding carnivore and animal-based diets often rely on selective citation of mechanistic studies or anecdotal success stories, while downplaying uncertainty, heterogeneity of responses, and the absence of long-term outcome data. From a scientific perspective, the popularity of a dietary model should not be conflated with evidentiary strength, and heightened visibility may, paradoxically, increase the risk of overinterpretation rather than clarify physiological plausibility.

From an exercise physiology perspective, the central question remains whether highly restrictive dietary models are compatible with the realities of contemporary training. In disciplines characterized by high relative intensity, high training volume, or rapid recovery demands, limited carbohydrate availability may constitute a meaningful constraint on adaptation [85]. Under such conditions, potential metabolic benefits do not compensate for functional losses [65]. The practical significance of these limitations differs across sporting disciplines. In ultra-endurance contexts, where exercise intensity remains relatively low and stable for prolonged periods, increased fat oxidation may in some cases align with the dominant energetic demands of the event. However, even in such settings, evidence does not consistently demonstrate superior performance outcomes compared with carbohydrate-supported approaches. By contrast, in sports requiring repeated surges in intensity, rapid accelerations, substantial glycolytic contribution, or dense competition schedules, restricted carbohydrate availability is more likely to constrain performance capacity, training quality, and recovery. This distinction underscores the importance of interpreting restrictive dietary strategies not as universally applicable models, but as interventions whose relevance depends strongly.

To make these sport-specific distinctions more explicit, Table 3 summarizes representative performance-related findings from studies on ketogenic or low-carbohydrate diets across different exercise contexts. Because no direct experimental studies on carnivore diets in athletes are currently available, the table synthesizes findings from the closest available physiological models reported in the literature on the metabolic structure of the sporting task.

At the same time, a critical evaluation of carnivore- and animal-based diets should not lead to their wholesale dismissal as research objects. Under tightly controlled conditions, restrictive dietary interventions can serve as experimental tools to explore the limits of human metabolic adaptation [86]. However, any potential implementation requires precise specification of intervention goals and duration, as well as systematic monitoring of health parameters [87].

In light of these considerations, carnivore and animal-based diets should be regarded not as alternatives to established sports nutrition models, but as extreme cases within a broader continuum of dietary strategies. Their scientific relevance lies primarily in delineating the boundaries of metabolic adaptation and exposing the limitations of simplified nutritional narratives. Accordingly, future research should focus not on confirming their “effectiveness,” but on precisely defining the contexts in which they may be tolerated or potentially useful.

## 7. Research Perspectives on Animal-Derived Dietary Models in Sport: Toward Precision Nutrition

### 7.1. Introducing a Developmental Perspective

The preceding analysis of carnivore- and animal-based diets in sport has primarily examined limited empirical evidence and identified potential health risks associated with their restrictive nature. This perspective was justified early in the development of this research area, when the primary objective was to distinguish hypotheses and anecdotal narratives from knowledge grounded in scientific data. However, further progress requires moving beyond simplistic dichotomies such as “safe versus unsafe” or “effective versus ineffective” and shifting emphasis toward a more nuanced, systems-oriented approach.

A prospective perspective on carnivore and animal-based diets does not entail forecasting their widespread adoption or unconditionally rejecting these dietary models. Rather, it aims to conceptually organize plausible research trajectories that could transform current controversies into a coherent, empirically grounded field of knowledge. This requires reframing the question from “do these diets work?” to an analysis of the conditions under which specific dietary interventions may be neutral, potentially beneficial, or, conversely, incompatible with the training and health requirements of athletes. Such a shift aligns with broader developments in precision nutrition and personalized medicine, in which variability in biological responses takes center stage rather than the pursuit of universally optimal dietary models.

### 7.2. Conceptual Evolution: From Ideology to Dynamic Protocols

Current perceptions of carnivore and animal-based diets in sport discourse often appear rigid and ideologically driven. These models are frequently presented as an “all-or-nothing” alternative to conventional dietary recommendations, which promotes simplification and polarization. From a developmental perspective, it appears far more plausible that these diets will transition from being treated as enduring dietary patterns toward being adapted as flexible, time-limited protocols. One possible direction involves cyclic management of energy substrates. In this framework, periodic implementation of very low-carbohydrate diets could be used to induce specific metabolic adaptations, such as enhanced fat oxidation capacity or improved glycemic control, followed by controlled carbohydrate reintroduction to optimize exercise performance. Such an approach could, in principle, combine putative adaptive benefits with the practical demands of high-intensity training. Similar concepts have previously been proposed in the literature on carbohydrate periodization in sport, where strategic manipulation of carbohydrate availability is used to stimulate specific metabolic adaptations while preserving the capacity for high-intensity training and competition (Impey et al., 2018 [22]). Alternatively, restrictive animal-derived dietary models may be conceptualized as short-term diagnostic tools. In this context, an elimination diet would not constitute an end in itself but rather a transitional phase to identify individual food intolerances, gastrointestinal issues, or adverse metabolic responses. Such an application shifts carnivore- and animal-based diets from the domain of ideology to that of controlled, function-oriented interventions.

### 7.3. Nutrition Personalization: The Role of Genetics, Metabolism, and the Microbiome

The development of research on carnivore and animal-based diets in sport is inseparable from advances in nutrigenomics, metabolomics, and gut microbiome science. It is increasingly evident that individual genetic traits, baseline metabolic profile, and the composition and function of the gut microbiota may substantially determine physiological responses to a given dietary model. In the future, it may be possible to more precisely identify athletes who tolerate periodic restriction of plant foods relatively well and those for whom such interventions entail an elevated risk of metabolic, gastrointestinal, or hormonal disturbances. Baseline microbiome profiling may become a key inclusion criterion for interventional studies, thereby reducing the risk of adverse health effects. At the same time, it should be emphasized that nutrition personalization does not imply arbitrariness or an absence of methodological constraints. On the contrary, it requires rigorous monitoring protocols that encompass objective physiological markers and subjective indices of well-being, cognitive function, and the individual’s relationship with food. It is important to emphasize that, despite its conceptual appeal, precision nutrition in sport remains an emerging research framework rather than an established applied paradigm. Current limitations include incomplete mechanistic understanding, limited availability of validated biomarkers, and substantial logistical and economic barriers to implementation. Consequently, the relevance of precision-based approaches to carnivore and animal-based diets should, at present, be viewed primarily as a direction for future investigation rather than as a basis for routine nutritional decision-making in athletic practice.

### 7.4. Emerging Methodological Frameworks in Sports Nutrition Research

Addressing the complex questions surrounding carnivore and animal-based diets requires moving beyond simplified research designs. Future studies should incorporate deep phenotyping of participants, including genetic, metabolic, hormonal, and microbiological assessments. Only such an approach will enable precise interpretation of observed effects and discrimination between potentially beneficial adaptations and potentially adverse consequences. A growing role may also be played by technologies that enable continuous monitoring of physiological parameters under real-world conditions, such as continuous glucose monitoring systems, heart rate variability analysis, and advanced tools for quantifying training load. Integrating data across multiple biological levels may allow detection of early signals of metabolic strain or impaired recovery before measurable declines in performance or overt health problems occur. In parallel, the development of long-term observational studies involving athletes adhering to different dietary models appears warranted. Such projects could provide data on the durability of adaptive effects and the long-term influence of restrictive diets on health and athletic career trajectories.

### 7.5. Ethical, Practical, and Environmental Challenges

Analysis of the developmental prospects of animal-derived dietary models in sport must account for ethical, psychological, and environmental considerations. Dietary patterns characterized by high consumption of animal products are associated with substantial environmental burdens and high economic costs, which limit their feasibility for widespread implementation in applied sport settings. Research examining the environmental footprint of dietary patterns consistently shows that diets rich in animal-derived foods are associated with higher greenhouse gas emissions, land use, and water demand than more plant-inclusive dietary patterns [88,89]. Beyond ethical considerations, the heavy reliance on animal-derived foods also poses practical constraints on feasibility, accessibility, and sustainability in applied sport settings. Diets characterized by elevated consumption of animal products are associated with higher economic costs and greater environmental burden, which may limit their scalability beyond highly controlled or individualized contexts. From a translational perspective, these factors further restrict the applicability of carnivore and animal-based dietary models as broadly implementable strategies within organized sport systems.

### 7.6. Prospective Synthesis

Future experimental studies investigating restrictive dietary strategies in athletes should incorporate clearly defined safety criteria and predefined thresholds for discontinuing the intervention. A developmental perspective on research into carnivore and animal-based diets in sport does not yield simple conclusions or unequivocal recommendations. It is far more likely that future investigations will contribute to a clearer understanding of the limits of human metabolic adaptation and of the role of specific dietary components in shaping health and physical performance.

Within this framework, restrictive animal-derived dietary models may come to function less as endpoint nutritional strategies and more as research and clinical tools that enable the design of more precise, context-sensitive nutritional interventions. The ultimate objective remains the development of flexible, safe, and evidence-informed dietary protocols tailored to the athlete’s individual characteristics, sporting discipline, and training stage. However, extremely elimination-based diets may adversely affect athletes’ relationship with food and increase the risk of restrictive eating behaviors, particularly in elite sport populations where pressure to optimize body mass and performance is pronounced (Table 4). For this reason, any future research and potential applications of such diets should incorporate a psycho-dietetic support component.

In this context, emerging precision nutrition frameworks may offer a promising methodological approach for evaluating highly restrictive dietary models in athletes. Rather than assuming uniform physiological responses, future research could examine individual variability through integrated monitoring of metabolic biomarkers, body composition changes, and sport-specific performance outcomes. Such individualized experimental approaches may help determine whether certain athletes exhibit favorable metabolic adaptations while others experience adverse responses to restrictive dietary patterns. This perspective highlights the importance of personalized monitoring when investigating unconventional dietary strategies in sport.

## 8. Conclusions

The conclusions drawn in this review should be interpreted in the context of important limitations in the currently available evidence, including the absence of randomized controlled trials directly examining carnivore diets in athletic populations and the reliance on indirect evidence derived from studies of related dietary models.

Analysis of the available literature indicates that carnivore and animal-based diets cannot presently be regarded as fully evidence-based nutritional strategies for athletic populations. The absence of high-quality interventional studies conducted under real athletic training conditions precludes definitive recommendations regarding their efficacy, safety, and long-term effects on health and exercise performance. An additional consideration when interpreting these conclusions is the substantial heterogeneity of physiological and performance demands across sporting disciplines. The metabolic constraints, training structure, and recovery requirements of endurance, strength, power, and mixed-modal sports differ markedly, limiting the external validity of generalized dietary conclusions. As a result, any inference regarding the applicability of carnivore or animal-based diets should be framed within clearly specified sporting contexts rather than extrapolated across the athlete population as a whole.

Evidence from research on very low-carbohydrate and ketogenic diets suggests that carbohydrate restriction can induce marked metabolic adaptations, particularly increased fat oxidation capacity. However, these adaptations are selective and context-dependent, and their potential utility appears limited to specific forms of low- to moderate-intensity exercise. Substantial evidence indicates that strategies involving chronic carbohydrate restriction may impose meaningful physiological constraints in disciplines that require high relative exercise intensity, rapid recovery between training sessions, and repeated high-power outputs.

A central conclusion of this review is the disconnect between observed metabolic changes and their actual relevance to athletic outcomes. Improvements in selected markers of metabolic health or biochemical adaptation do not necessarily translate into enhanced performance and, in some cases, may co-occur with deterioration in parameters critical for training and competitive success. This pattern underscores the need for cautious interpretation of findings and avoidance of simplified narratives built around isolated physiological mechanisms.

A major limitation of carnivore and animal-based diets is their extremely elimination-based character. Long-term exclusion of plant foods is associated with an increased risk of micronutrient inadequacy, adverse shifts in the gut microbiome, and disturbances in lipid metabolism among individuals at risk. Moreover, highly restrictive dietary models may affect the relationship with food and promote restrictive eating behaviors, which constitute a significant threat to mental health and long-term athletic development in elite sport populations.

Given the current evidence, carnivorous and animal-based diets cannot be recommended as safe or optimal nutritional strategies for the general athlete population. It should also be emphasized that no randomized controlled trials have directly examined carnivore diets in athletic populations. Consequently, a substantial proportion of the interpretative framework presented in this review relies on indirect evidence derived from related dietary models, particularly ketogenic and low-carbohydrate interventions. Although such studies offer valuable physiological insight, they should not be interpreted as direct evidence of efficacy, safety, or performance effects specific to carnivore diets in sport. This limitation should remain central to any interpretation of the present conclusions. The increasing popularity of these diets in athletic communities outpaces the available scientific evidence and is driven largely by anecdotal accounts and overinterpretation of indirect research findings. This phenomenon reflects a broader challenge in sports nutrition research: the pursuit of universal solutions in a domain characterized by substantial interindividual variability in biological responses.

At the same time, wholesale dismissal of carnivore- and animal-based diets as subjects of scientific inquiry does not appear justified. These models may possess explanatory value as research tools, facilitating exploration of the limits of metabolic adaptation and identification of determinants of individual tolerance to restrictive dietary interventions. However, any potential application should be confined to tightly controlled, short-term research or diagnostic protocols accompanied by rigorous monitoring of health parameters.

Ultimately, this review suggests that the future of research on animal-derived dietary models in sport lies not in identifying another alternative to established sports nutrition paradigms, but in advancing precise, context-specific, and individually tailored nutritional strategies. Elucidating the boundaries of metabolic adaptation, the interactions among diet, training, and health, and the role of interindividual variability remain central challenges for future research and applied practice.

## Figures and Tables

**Table 1:** Comparative characteristics of carnivore, animal-based, VLCKD, and conventional sports diets.

Dietary Model	Core Foods	Excluded Foods	Typical Carbohydrate Intake	Typical Protein Intake	Predominant Metabolic State	Key References
Carnivore diet	Meat, organ meats, fish, eggs, animal fats (sometimes dairy)	All plant foods	~0–5% of energy	High (often >2.5 g/kg/day)	Nutritional ketosis (variant-dependent)	[12,13]
Animal-based diet	Animal foods + fruit, honey, selected dairy	Grains, legumes, seed oils, and many vegetables	Low–moderate (variant-dependent)	High	Mixed substrate utilization (ketosis uncommon in higher-CHO variants)	[31,35,36,39]
VLCKD	High-fat foods, moderate protein, minimal carbohydrates	Grains, sugars, starches	<10% energy (<50 g/day)	Moderate	Adaptive nutritional ketosis	[7,9]
LCHF	High fat, moderate protein, restricted carbohydrates	Refined grains, sugars, starches	<10–25% of energy	Moderate–high	Increased fat oxidation; variable ketosis	[40,41]
Conventional sports diet	Mixed plant and animal foods	No systematic exclusions	Moderate–high (periodized)	Goal-dependent	Periodized carbohydrate availability; metabolic flexibility	[1,3]

Abbreviations: LCHF—low-carbohydrate, high-fat diet. VLCKD—very-low-carbohydrate ketogenic diet. CHO—carbohydrates.

**Table 2:** Postulated benefits of carnivore and animal-based diets versus the strength of evidence.

Postulated Effect	Theoretical Rationale	Indirect Evidence (Related Diets)	Direct Evidence (Carnivore/Animal-Based)	Evidence Strength	Key References
Reduced systemic inflammation	Removal of specific plant-derived bioactive compounds (e.g., antinutrients)	Partial (VLCKD studies)	None	Low	[12,60]
Stable energy availability	Reduced glycaemic variability and increased fat reliance	Yes	None	Low–moderate	[9,38]
Improved body composition	High protein intake and ketosis-mediated metabolic shifts	Yes	None	Moderate (indirect)	[22,61]
Enhanced recovery	Reduced exposure to poorly tolerated dietary components; potential modulation of GI stress	Conflicting	None	Very low	[62,63]
Improved endurance efficiency	Increased fat oxidation and altered substrate utilization	Yes	None	Low	[64,65]
High-intensity performance	Reduced carbohydrate availability	Negative findings	None	Low (evidence against)	[40,66]

**Table 3:** Representative performance-related findings from ketogenic and low-carbohydrate dietary interventions across sport contexts.

Sport Context	Representative Population or Model	Dietary Model	Main Physiological Adaptation	Reported Performance Implication	Key References
Ultra-endurance/prolonged endurance exercise	Keto-adapted ultra-endurance athletes	Ketogenic/very-low-carbohydrate diet	Markedly increased fat oxidation capacity	Metabolic adaptation clearly demonstrated, but without consistent evidence of superior competitive performance	[7,8]
Elite endurance competition	Elite race walkers	Low-carbohydrate, high-fat diet	Increased fat utilization	Impaired exercise economy and no improvement in performance benefit from training	[9]
Endurance training with chronic carbohydrate restriction	Elite race walkers	Ketogenic low-carbohydrate, high-fat diet	Sustained carbohydrate restriction during training	Diminished training quality under elite training conditions	[40]
High-intensity or variable-intensity endurance contexts	Endurance-trained athletes	Periodized carbohydrate restriction/low-carbohydrate strategies	Increased metabolic stress associated with reduced carbohydrate availability	Performance outcomes generally unfavorable when high-intensity demands are substantial	[44,66]
Sports requiring repeated high-power output or glycolytic contribution	Applied mixed or intermittent sport interpretation based on low-carbohydrate literature	Ketogenic/low-carbohydrate models	Reduced glycogen availability and constrained glycolytic support	Likely functional limitations in repeated high-intensity efforts and recovery-dependent sport settings	[42,66,77]

Note: Because no direct experimental studies currently examine carnivore diets in athletic populations, the findings summarized here are derived from ketogenic or low-carbohydrate dietary interventions discussed in the present review. These studies were included as the closest available physiological analogues, not as direct evidence of the efficacy of carnivore diets in sport. The table is intended to improve contextual clarity rather than to imply equivalence between carnivore diets and the related dietary models from which these findings are derived.

**Table 4:** Potential risks and unresolved research gaps.

Domain	Potential Concern	Current Evidence Status	Relevance for Athletes	Key References
Gut microbiota	Absence of dietary fiber; reduced short-chain fatty acid (SCFA) production	Mechanistic and observational evidence; no athlete-specific intervention trials	Recovery, immune resilience, gut integrity during high training loads	[90,91]
Micronutrient adequacy	Risk of low intake of vitamin C, K1, folate, magnesium, and phytochemicals	Limited direct data; extrapolated from elimination-based dietary models	Long-term health, antioxidant defense, connective tissue integrity	[92,93]
Lipid profile	LDL-C elevation in lean hyper-responders; inter-individual variability	Heterogeneous responses; subgroup-specific elevations documented	Cardiovascular risk stratification in susceptible individuals	[94,95]
High-intensity capacity	Reduced glycolytic flux and glycogen availability	Consistently negative findings in high-intensity endurance contexts	Power output, repeat sprint ability, training quality	[40,96]
Long-term safety	Lack of ≥12-month controlled trials in athletes	Major research gap; absence of longitudinal safety data	Critical for multi-year athletic careers	[97,98]

## Data Availability

No new data were created or analyzed in this study. Data sharing is not applicable to this article.

## Supplementary Materials

The following supporting information can be downloaded at: https://www.mdpi.com/article/10.3390/nu18060998/s1, Table S1: General structure of the literature search strategy.

## References

[1] Burke L.M., Hawley J.A., Wong S.H.S., Jeukendrup A.E. (2011). Carbohydrates for Training and Competition. J. Sports Sci..

[2] Bartlett J.D., Hawley J.A., Morton J.P. (2015). Carbohydrate Availability and Exercise Training Adaptation: Too Much of a Good Thing?. Eur. J. Sport Sci..

[3] Thomas D.T., Erdman K.A., Burke L.M. (2016). Position of the Academy of Nutrition and Dietetics, Dietitians of Canada, and the American College of Sports Medicine: Nutrition and Athletic Performance. J. Acad. Nutr. Diet..

[4] Jeukendrup A.E. (2017). Periodized Nutrition for Athletes. Sports Med..

[5] Jäger R., Kerksick C.M., Campbell B.I., Cribb P.J., Wells S.D., Skwiat T.M., Purpura M., Ziegenfuss T.N., Ferrando A.A., Arent S.M. (2017). International Society of Sports Nutrition Position Stand: Protein and Exercise. J. Int. Soc. Sports Nutr..

[6] Phinney S.D., Bistrian B.R., Evans W.J., Gervino E., Blackburn G.L. (1983). The Human Metabolic Response to Chronic Ketosis without Caloric: Preservation of Submaximal Exercise Capability with reduced Carbohydrate Oxidation. Metabolism.

[7] Volek J.S., Noakes T., Phinney S.D. (2015). Rethinking Fat as a Fuel for Endurance Exercise. Eur. J. Sport Sci..

[8] Volek J.S., Freidenreich D.J., Saenz C., Kunces L.J., Creighton B.C., Bartley J.M., Davitt P.M., Munoz C.X., Anderson J.M., Maresh C.M. (2016). Metabolic Characteristics of Keto-Adapted Ultra-Endurance Runners. Metabolism.

[9] Burke L.M., Ross M.L., Garvican-Lewis L.A., Welvaert M., Heikura I.A., Forbes S.G., Mirtschin J.G., Cato L.E., Strobel N., Sharma A.P. (2017). Low Carbohydrate, High Fat Diet Impairs Exercise Economy and Negates the Performance Benefit from Intensified Training in Elite Race Walkers. J. Physiol..

[10] McSwiney F.T., Wardrop B., Hyde P.N., Lafountain R.A., Volek J.S., Doyle L. (2018). Keto-Adaptation Enhances Exercise Performance and Body Composition Responses to Training in Endurance Athletes. Metabolism.

[11] Mata F., Valenzuela P.L., Gimenez J., Tur C., Ferreria D., Domínguez R., Sanchez-Oliver A.J., Sanz J.M.M. (2019). Carbohydrate Availability and Physical Performance: Physiological Overview and Practical Recommendations. Nutrients.

[12] O’Hearn A. (2020). Can a Carnivore Diet Provide All Essential Nutrients?. Curr. Opin. Endocrinol. Diabetes Obes..

[13] Lennerz B.S., Mey J.T., Henn O.H., Ludwig D.S. (2021). Behavioral Characteristics and Self-Reported Health Status among 2029 Adults Consuming a “Carnivore Diet.”. Curr. Dev. Nutr..

[14] Dowis K., Banga S. (2021). The Potential Health Benefits of the Ketogenic Diet: A Narrative Review. Nutrients.

[15] Burke L.M. (2021). Ketogenic Low-CHO, High-Fat Diet: The Future of Elite Endurance Sport?. J. Physiol..

[16] Ferguson L.R., De Caterina R., Görman U., Allayee H., Kohlmeier M., Prasad C., Choi M.S., Curi R., De Luis D.A., Gil Á. (2016). Guide and Position of the International Society of Nutrigenetics/Nutrigenomics on Personalised Nutrition: Part 1—Fields of Precision Nutrition. J. Nutr. Nutr..

[17] Mardinoglu A., Wu H., Bjornson E., Zhang C., Hakkarainen A., Räsänen S.M., Lee S., Mancina R.M., Bergentall M., Pietiläinen K.H. (2018). An Integrated Understanding of the Rapid Metabolic Benefits of a Carbohydrate-Restricted Diet on Hepatic Steatosis in Humans. Cell Metab..

[18] Zeevi D., Korem T., Zmora N., Israeli D., Rothschild D., Weinberger A., Ben-Yacov O., Lador D., Avnit-Sagi T., Lotan-Pompan M. (2015). Personalized Nutrition by Prediction of Glycemic Responses. Cell.

[19] Ordovas J.M., Ferguson L.R., Tai E.S., Mathers J.C. (2018). Personalised Nutrition and Health. BMJ.

[20] Greenhalgh T., Thorne S., Malterud K. (2018). Time to Challenge the Spurious Hierarchy of Systematic over Narrative Reviews?. Eur. J. Clin. Investig..

[21] Moher D., Liberati A., Tetzlaff J., Altman D.G., Antes G., Atkins D., Barbour V., Barrowman N., Berlin J.A., Clark J. (2009). Preferred Reporting Items for Systematic Reviews and Meta-Analyses: The PRISMA Statement. PLoS Med..

[22] Impey S.G., Hearris M.A., Hammond K.M., Bartlett J.D., Louis J., Close G.L., Morton J.P. (2018). Fuel for the Work Required: A Theoretical Framework for Carbohydrate Periodization and the Glycogen Threshold Hypothesis. Sports Med..

[23] Calder P.C., Carr A.C., Gombart A.F., Eggersdorfer M. (2020). Optimal Nutritional Status for a Well-Functioning Immune System Is an Important Factor to Protect against Viral Infections. Nutrients.

[24] Satija A., Hu F.B. (2018). Plant-Based Diets and Cardiovascular Health. Trends Cardiovasc. Med..

[25] Ioannidis J.P.A. (2018). Meta-Research: Why Research on Research Matters. PLoS Biol..

[26] Subar A.F., Freedman L.S., Tooze J.A., Kirkpatrick S.I., Boushey C., Neuhouser M.L., Thompson F.E., Potischman N., Guenther P.M., Tarasuk V. (2015). Addressing Current Criticism Regarding the Value of Self-Report Dietary Data. J. Nutr..

[27] Hernán M.A., Hsu J., Healy B. (2019). A Second Chance to Get Causal Inference Right: A Classification of Data Science Tasks. CHANCE.

[28] Byndloss M.X., Olsan E.E., Rivera-Chávez F., Tiffany C.R., Cevallos S.A., Lokken K.L., Torres T.P., Byndloss A.J., Faber F., Gao Y. (2017). Microbiota-Activated PPAR-γ Signaling Inhibits Dysbiotic Enterobacteriaceae Expansion. Science.

[29] Johnston B.C., Kanters S., Bandayrel K., Wu P., Naji F., Siemieniuk R.A., Ball G.D.C., Busse J.W., Thorlund K., Guyatt G. (2014). Comparison of Weight Loss among Named Diet Programs in Overweight and Obese Adults: A Meta-Analysis. JAMA.

[30] Gibson A.A., Seimon R.V., Lee C.M.Y., Ayre J., Franklin J., Markovic T.P., Caterson I.D., Sainsbury A. (2015). Do Ketogenic Diets Really Suppress Appetite? A Systematic Review and Meta-Analysis. Obes. Rev..

[31] Feinman R.D., Pogozelski W.K., Astrup A., Bernstein R.K., Fine E.J., Westman E.C., Accurso A., Frassetto L., Gower B.A., McFarlane S.I. (2015). Dietary Carbohydrate Restriction as the First Approach in Diabetes Management: Critical Review and Evidence Base. Nutrition.

[32] Paoli A. (2014). Ketogenic Diet for Obesity: Friend or Foe?. Int. J. Environ. Res. Public Health.

[33] Paoli A., Rubini A., Volek J.S., Grimaldi K.A. (2013). Beyond Weight Loss: A Review of the Therapeutic Uses of Very-Low-Carbohydrate (Ketogenic) Diets. Eur. J. Clin. Nutr..

[34] Astrup A., Magkos F., Bier D.M., Brenna J.T., de Oliveira Otto M.C., Hill J.O., King J.C., Mente A., Ordovas J.M., Volek J.S. (2020). Saturated Fats and Health: A Reassessment and Proposal for Food-Based Recommendations: JACC State-of-the-Art Review. J. Am. Coll. Cardiol..

[35] Westman E.C., Feinman R.D., Mavropoulos J.C., Vernon M.C., Volek J.S., Wortman J.A., Yancy W.S., Phinney S.D. (2007). Low-Carbohydrate Nutrition and Metabolism. Am. J. Clin. Nutr..

[36] Paoli A., Bianco A., Grimaldi K.A. (2015). The Ketogenic Diet and Sport: A Possible Marriage?. Exerc. Sport Sci. Rev..

[37] Volek J.S., Volk B.M., Phinney S.D. (2012). The Twisted Tale of Saturated Fat. Lipid Technol..

[38] Noakes T., Volek J.S., Phinney S.D. (2014). Low-Carbohydrate Diets for Athletes: What Evidence?. Br. J. Sports Med..

[39] Cordain L., Miller J.B., Eaton S.B., Mann N., Holt S.H.A., Speth J.D. (2000). Plant-Animal Subsistence Ratios and Macronutrient Energy Estimations in Worldwide Hunter-Gatherer Diets. Am. J. Clin. Nutr..

[40] McKay A.K.A., Ross M.L.R., Tee N., Sharma A.P., Leckey J.J., Burke L.M. (2023). Adherence to a Ketogenic Low-Carbohydrate, High-Fat Diet Is Associated With Diminished Training Quality in Elite Racewalkers. Int. J. Sports Physiol. Perform..

[41] Cao J., Lei S., Wang X., Cheng S. (2021). The Effect of a Ketogenic Low-Carbohydrate, High-Fat Diet on Aerobic Capacity and Exercise Performance in Endurance Athletes: A Systematic Review and Meta-Analysis. Nutrients.

[42] Hargreaves M., Spriet L.L. (2020). Skeletal Muscle Energy Metabolism during Exercise. Nat. Metab..

[43] Shaw D.M., Merien F., Braahuis A., Maunder E.D., Dulson D.K. (2019). Effect of a Ketogenic Diet on Submaximal Exercise Capacity and Efficiency in Runners. Med. Sci. Sports Exerc..

[44] Gejl K.D., Nybo L. (2021). Performance Effects of Periodized Carbohydrate Restriction in Endurance Trained Athletes—A Systematic Review and Meta-Analysis. J. Int. Soc. Sports Nutr..

[45] Durkalec-Michalski K., Domagalski A., Główka N., Kamińska J., Szymczak D., Podgórski T. (2022). Effect of a Four-Week Vegan Diet on Performance, Training Efficiency and Blood Biochemical Indices in CrossFit-Trained Participants. Nutrients.

[46] Lis D.M., Stellingwerff T., Kitic C.M., Fell J.W., Ahuja K.D.K. (2018). Low FODMAP: A Preliminary Strategy to Reduce Gastrointestinal Distress in Athletes. Med. Sci. Sports Exerc..

[47] Spriet L.L. (2014). New Insights into the Interaction of Carbohydrate and Fat Metabolism during Exercise. Sports Med..

[48] Bergström J., Hermansen L., Hultman E., Saltin B. (1967). Diet, Muscle Glycogen and Physical Performance. Acta Physiol. Scand..

[49] Morton R.W., Murphy K.T., McKellar S.R., Schoenfeld B.J., Henselmans M., Helms E., Aragon A.A., Devries M.C., Banfield L., Krieger J.W. (2017). A Systematic Review, Meta-Analysis and Meta-Regression of the effect of Protein Supplementation on Resistance Training-Induced Gains in muscle Mass and Strength in Healthy Adults. Br. J. Sports Med..

[50] Antonio J., Peacock C.A., Ellerbroek A., Fromhoff B., Silver T. (2014). The Effects of Consuming a High Protein Diet (4.4 g/Kg/d) on Body Composition in Resistance-Trained Individuals. J. Int. Soc. Sports Nutr..

[51] Escobar K.A., Vandusseldorp T.A., Kerksick C.M. (2016). Carbohydrate Intake and Resistance-Based Exercise: Are Current Recommendations Reflective of Actual Need?. Br. J. Nutr..

[52] Hyde P.N., Sapper T.N., Crabtree C.D., LaFountain R.A., Bowling M.L., Buga A., Fell B., McSwiney F.T., Dickerson R.M., Miller V.J. (2019). Dietary Carbohydrate Restriction Improves Metabolic Syndrome Independent of Weight Loss. JCI Insight.

[53] Gershuni V.M., Yan S.L., Medici V. (2018). Nutritional Ketosis for Weight Management and Reversal of Metabolic Syndrome. Curr. Nutr. Rep..

[54] Bowtell J., Kelly V. (2019). Fruit-Derived Polyphenol Supplementation for Athlete Recovery and Performance. Sports Med..

[55] Brooks G.A. (2020). Lactate as a Fulcrum of Metabolism. Redox Biol..

[56] Hallberg S.J., McKenzie A.L., Williams P.T., Bhanpuri N.H., Peters A.L., Campbell W.W., Hazbun T.L., Volk B.M., McCarter J.P., Phinney S.D. (2018). Effectiveness and Safety of a Novel Care Model for the Management of Type 2 Diabetes at 1 Year: An Open-Label, Non-Randomized, Controlled Study. Diabetes Ther..

[57] Hall K.D., Chen K.Y., Guo J., Lam Y.Y., Leibel R.L., Mayer L.E.S., Reitman M.L., Rosenbaum M., Smith S.R., Walsh B.T. (2016). Energy Expenditure and Body Composition Changes after an Isocaloric Ketogenic Diet in Overweight and Obese Men. Am. J. Clin. Nutr..

[58] David L.A., Maurice C.F., Carmody R.N., Gootenberg D.B., Button J.E., Wolfe B.E., Ling A.V., Devlin A.S., Varma Y., Fischbach M.A. (2014). Diet Rapidly and Reproducibly Alters the Human Gut Microbiome. Nature.

[59] Sonnenburg E.D., Smits S.A., Tikhonov M., Higginbottom S.K., Wingreen N.S., Sonnenburg J.L. (2016). Diet-Induced Extinctions in the Gut Microbiota Compound over Generations. Nature.

[60] Calder P.C., Ahluwalia N., Brouns F., Buetler T., Clement K., Cunningham K., Esposito K., Jönsson L.S., Kolb H., Lansink M. (2011). Dietary Factors and Low-Grade Inflammation in Relation to Overweight and Obesity. Br. J. Nutr..

[61] Mettler S., Mitchell N., Tipton K. (2010). Increased Protein Intake Reduces Lean Body Mass Loss during Weight in Athletes. Med. Sci. Sports Exerc..

[62] Nieman D.C. (2012). Clinical Implications of Exercise Immunology. J. Sport Health Sci..

[63] Powers S.K., Nelson W.B., Hudson M.B. (2011). Exercise-Induced Oxidative Stress in Humans: Cause and Consequences. Free Radic. Biol. Med..

[64] Prins P., Noakes T., Buxton J., Welton G., Raabe A., Scott K., Atwell A., Haley S., Esbenshade N., Abraham J. (2023). High Fat Diet Improves Metabolic Flexibility during Progressive to Exhaustion (VO 2 Max Testing) and during 5km Running Time. Biol. Sport.

[65] Cao W., He Y., Fu R., Chen Y., Yu J., He Z. (2025). A Review of Carbohydrate Supplementation Approaches and Strategies for Optimizing Performance in Elite Long-Distance Endurance. Nutrients.

[66] Leaf A., Rothschild J.A., Sharpe T.M., Sims S.T., Macias C.J., Futch G.G., Roberts M.D., Stout J.R., Ormsbee M.J., Aragon A.A. (2024). International Society of Sports Nutrition Position Stand: Ketogenic Diets. J. Int. Soc. Sports Nutr..

[67] Makki K., Deehan E.C., Walter J., Bäckhed F. (2018). The Impact of Dietary Fiber on Gut Microbiota in Host Health and Disease. Cell Host Microbe.

[68] Leeming E.R., Johnson A.J., Spector T.D., Roy C.I.L. (2019). Effect of Diet on the Gut Microbiota: Rethinking Intervention Duration. Nutrients.

[69] Costa R.J.S., Snipe R.M.J., Kitic C.M., Gibson P.R. (2017). Systematic Review: Exercise-Induced Gastrointestinal Syndrome—Implications for Health and Intestinal Disease. Aliment. Pharmacol. Ther..

[70] Aune D., Chan D.S.M., Lau R., Vieira R., Greenwood D.C., Kampman E., Norat T. (2011). Dietary Fibre, Whole Grains, and Risk of Colorectal Cancer: Systematic Review and Dose-Response Meta-Analysis of Prospective Studies. BMJ.

[71] Dinu M., Abbate R., Gensini G.F., Casini A., Sofi F. (2017). Vegetarian, Vegan Diets and Multiple Health Outcomes: A Systematic Review with Meta-Analysis of Observational Studies. Crit. Rev. Food Sci. Nutr..

[72] Melina V., Craig W., Levin S. (2016). Position of the Academy of Nutrition and Dietetics: Vegetarian Diets. J. Acad. Nutr. Diet..

[73] Turnlund J.R., Betschart A.A., Liebman M., Kretsch M.J., Sauberlich H.E. (1992). Vitamin B−6 Depletion Followed by Repletion with Animal- or plant-Source Diets and Calcium and Magnesium Metabolism in Young Women. Am. J. Clin. Nutr..

[74] Poortmans J.R., Dellalieux O. (2000). Do Regular High Protein Diets Have Potential Health Risks on Kidney in Athletes?. Int. J. Sport Nutr. Exerc. Metab..

[75] Antonio J., Ellerbroek A., Silver T., Vargas L., Peacock C. (2016). The Effects of a High Protein Diet on Indices of Health and Body—A Crossover Trial in Resistance-Trained Men. J. Int. Soc. Sports Nutr..

[76] Mountjoy M., Sundgot-Borgen J.K., Burke L.M., Ackerman K.E., Blauwet C., Constantini N., Lebrun C., Lundy B., Melin A.K., Meyer N.L. (2018). IOC Consensus Statement on Relative Energy Deficiency in Sport (RED-S): 2018 Update. Br. J. Sports Med..

[77] Burke L.M., Whitfield J. (2024). Ketogenic Diets Are Not Beneficial for Athletic Performance. Med. Sci. Sports Exerc..

[78] Kosinski C., Jornayvaz F.R. (2017). Effects of Ketogenic Diets on Cardiovascular Risk Factors: Evidence from Animal and Human Studies. Nutrients.

[79] Joyner M.J., Coyle E.F. (2008). Endurance Exercise Performance: The Physiology of Champions. J. Physiol..

[80] Brehm B.J., Seeley R.J., Daniels S.R., D’Alessio D.A. (2003). A Randomized Trial Comparing a Very Low Carbohydrate Diet and a Calorie-Restricted Low Fat Diet on Body Weight and Cardiovascular Risk Factors in Healthy Women. J. Clin. Endocrinol. Metab..

[81] Maughan R.J., Burke L.M., Dvorak J., Larson-Meyer D.E., Peeling P., Phillips S.M., Rawson E.S., Walsh N.P., Garthe I., Geyer H. (2018). IOC Consensus Statement: Dietary Supplements and the High-Performance Athlete. Br. J. Sports Med..

[82] Fatt S.J., George E., Hay P., Jeacocke N., Gotkiewicz E., Mitchison D. (2024). An Umbrella Review of Body Image Concerns, Disordered Eating, and Eating Disorders in Elite Athletes. J. Clin. Med..

[83] Ludwig D.S., Ebbeling C.B. (2018). The Carbohydrate-Insulin Model of Obesity. JAMA Intern. Med..

[84] Ostojic S.M., Øverby N.C. (2023). Editorial: Scientific Integrity in Nutritional Research. Front. Nutr..

[85] Diaz-Lara J., Prieto-Bellver G., Guadalupe-Grau A., Bishop D.J. (2025). Responses to Exercise with Low Carbohydrate Availability on Muscle Glycogen and Cell Signaling: A Systematic Review and Meta-Analysis. Sports Med..

[86] Nikparast A., Mirzaei P., Tadayoni Z.S., Asghari G. (2025). The Association Between Overall, Healthy, and Unhealthy Plant-Based Diet Index and Risk of Prediabetes and Type 2 Diabetes Mellitus: A Systematic Review and Dose-Response Meta-Analysis of Prospective Studies. Nutr. Rev..

[87] Andrews V., Zammit G., O’Leary F. (2023). Dietary Pattern, Food, and Nutritional Supplement Effects on Cognitive Outcomes in Mild Cognitive Impairment: A Systematic Review of Previous Reviews. Nutr. Rev..

[88] Aleksandrowicz L., Green R., Joy E.J.M., Smith P., Haines A. (2016). The Impacts of Dietary Change on Greenhouse Gas Emissions, Land Use, Water Use, and Health: A Systematic Review. PLoS ONE.

[89] Poore J., Nemecek T. (2018). Reducing Food’s Environmental Impacts through Producers and Consumers. Science.

[90] Sumi D., Suzuki Y. (2025). Gastrointestinal Function and Microbiota in Endurance Athletes. Front. Physiol..

[91] Zhang X., Li Y., Zhang F., Zhou G. (2025). Exercise and Diet Reshape Athletes’ Gut Microbiota: Countering Health Challenges in Athletes. Life.

[92] Richards J.D., Cori H., Rahn M., Finn K., Bárcena J., Kanellopoulos A.K., Péter S., Spooren A. (2025). Micronutrient Bioavailability: Concepts, Influencing Factors, and Strategies for Improvement. Front. Nutr..

[93] Everett S. (2025). Optimizing Performance Nutrition for Adolescent Athletes: A Review of Dietary Needs, Risks, and Practical Strategies. Nutrients.

[94] Vargas-Molina S., Murri M., Gonzalez-Jimenez A., Gómez-Urquiza J.L., Benítez-Porres J. (2024). Effects of the Ketogenic Diet on Strength Performance in Trained Men and Women: A Systematic Review and Meta-Analysis. Nutrients.

[95] Joo M., Moon S., Lee Y.S., Kim M.G. (2023). Effects of Very Low-Carbohydrate Ketogenic Diets on Lipid Profiles in Normal-Weight (Body Mass Index < 25 Kg/M^2^) Adults: A Meta-Analysis. Nutr. Rev..

[96] Sun K., Choi Y.T., Yu C.C.W., Nelson E.A.S., Goh J., Dai S., Hui L.L. (2025). The Effects of Ketogenic Diets and Ketone Supplements on the Aerobic Performance of Endurance Runners: A Systematic Review. Sports Health.

[97] Carpenter M., Brouner J., Spendiff O. (2025). Strategic Carbohydrate Feeding Improves Performance in Ketogenic Trained Athletes. Clin. Nutr..

[98] Ichikawa T., Okada H., Hironaka J., Nakajima H., Okamura T., Majima S., Senmaru T., Ushigome E., Nakanishi N., Hamaguchi M. (2024). Efficacy of Long-Term Low Carbohydrate Diets for Patients with Type 2 Diabetes: A Systematic Review and Meta-Analysis. J. Diabetes Investig..

