# Supplementary material for: Carnivore and Animal-Based Diets in Sport: A Critical Evaluation of Current Evidence and Future Perspectives for Precision Nutrition
Source: Nutrients. 2026 Mar 21;18(6):998. doi: 10.3390/nu18060998 (PMC13029088; doi:10.3390/nu18060998)
Supplement: Supplementary file 1 [file nutrients-18-00998-s001.zip › nutrients-4193721-supplementary.pdf]

Supplementary Table S1. General structure of the literature search strategy

Database	Core diet-related terms	Core sport/performance-related terms	General focus of retrieved literature
PubMed	“carnivore diet”, “animal-based diet”, “ketogenic diet”, “low-carbohydrate diet”	“athlete”, “exercise performance”, “endurance”, “strength”, “sports nutrition”, “metabolism”	Direct and indirect evidence related to dietary restriction, metabolism, and athletic performance
Scopus	“carnivore diet”, “animal-based diet”, “ketogenic diet”, “low-carbohydrate diet”	“athlete”, “exercise”, “sport”, “performance”, “training”, “metabolic adaptation”	Broad interdisciplinary coverage of sports nutrition and metabolic research
Web of Science	“carnivore diet”, “animal-based diet”, “ketogenic diet”, “low-carbohydrate diet”	“exercise performance”, “athletic performance”, “endurance”, “strength”, “recovery”	Conceptual, experimental, and review-based evidence relevant to sport and dietary physiology

Table note

The search strategy was structured to identify both direct evidence on carnivore and animal-based diets and indirect evidence from related dietary models, particularly ketogenic and low-carbohydrate interventions. Because the review was narrative rather than systematic, the table is intended to improve transparency rather than to represent a PRISMA-style screening framework.
